# Supplementary material for: Oscillatory multi-timescale mechanisms underlying audiovisual sequence prediction
Source: Imaging Neurosci (Camb). 2026 Jan 16;4:IMAG.a.1103. doi: 10.1162/IMAG.a.1103 (PMC12813862; doi:10.1162/IMAG.a.1103)
Supplement: Supplementary Material [file IMAG.a.1103_supp.pdf]

Title:

Oscillatory Multi-Timescale Mechanisms Underlying Audiovisual Sequence Prediction

Running title: Multi-Timescale Dynamics of Sequence Prediction

Peng Wang[a,*], Alexander Maÿe[a], Jonathan Daume[a,b], Gui Xue[c] and Andreas K. Engel[a]

[a] Department of Neurophysiology and Pathophysiology, University Medical Center Hamburg-Eppendorf, Martinistr. 52, 20246 Hamburg, Germany

[b] Department of Neurosurgery, Cedars-Sinai Medical Center, Los Angeles, CA, USA

[c] State Key Laboratory of Cognitive Neuroscience and Learning & IDG/McGovern Institute for Brain Research, Beijing Normal University, 100875, Beijing, China

[*] Correspondence address:

Peng Wang, Phone: +49-40-741054680, Fax: +49-40-741057126, E-mail: p.wang@uke.de

Department of Neurophysiology and Pathophysiology, University Medical Center Hamburg-Eppendorf, Martinistr. 52, 20246 Hamburg, Germany.

Appendices

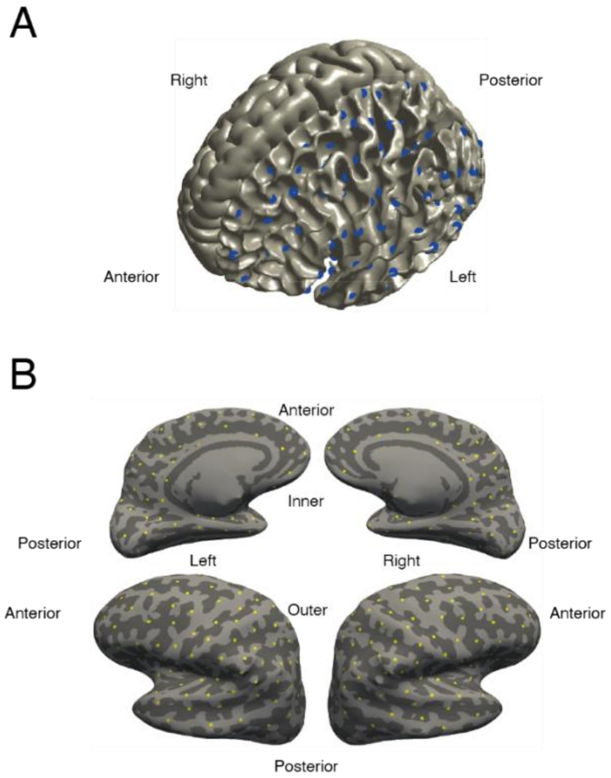

Fig. S1. Definition of the source space. (A) White matter (left hemisphere) and pial (right hemisphere) surfaces of the template brain. Source locations were evenly distributed on the surface of the white matter, as indicated by the blue dots in the left hemisphere. (B) The inflated view of the template brain. Source locations are indicated by the yellow dots on the inflated surface.

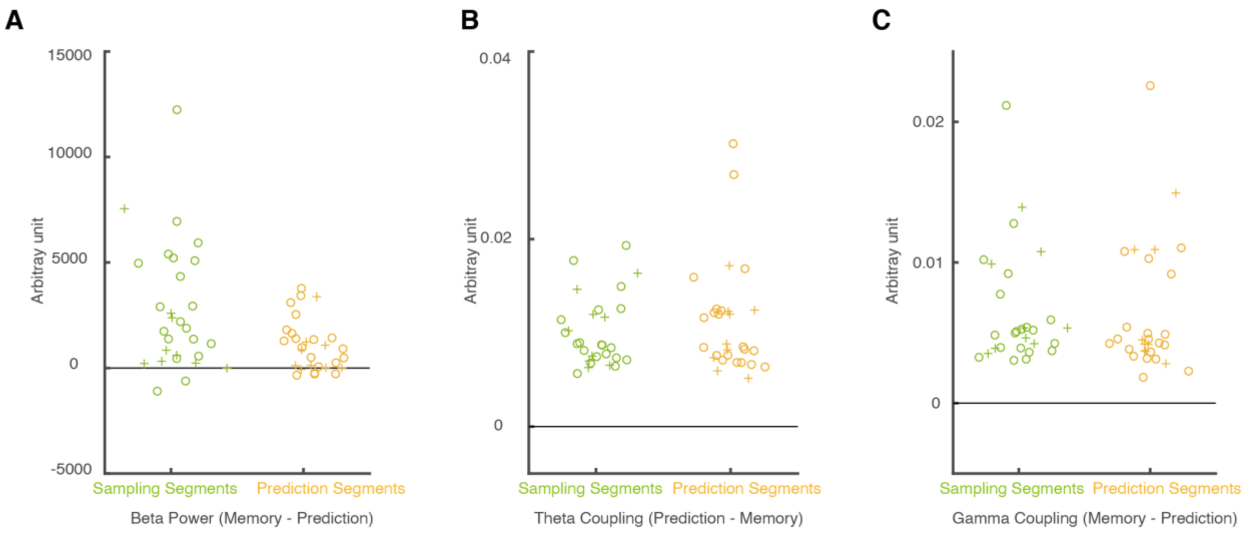

Fig. S2. Between-task differences in the identified clusters across control conditions.

The panels show differences between the prediction and memory tasks for (A) beta power (memory – prediction), (B) theta coupling (prediction – memory), and (C) gamma coupling (memory – prediction). The subtraction order was chosen to ensure positive values, facilitating easier comparison across different measures. Results are shown separately for sampling segments and prediction segments. Each symbol represents a participant, with two different control conditions. Horizontal black lines indicate zero differences.

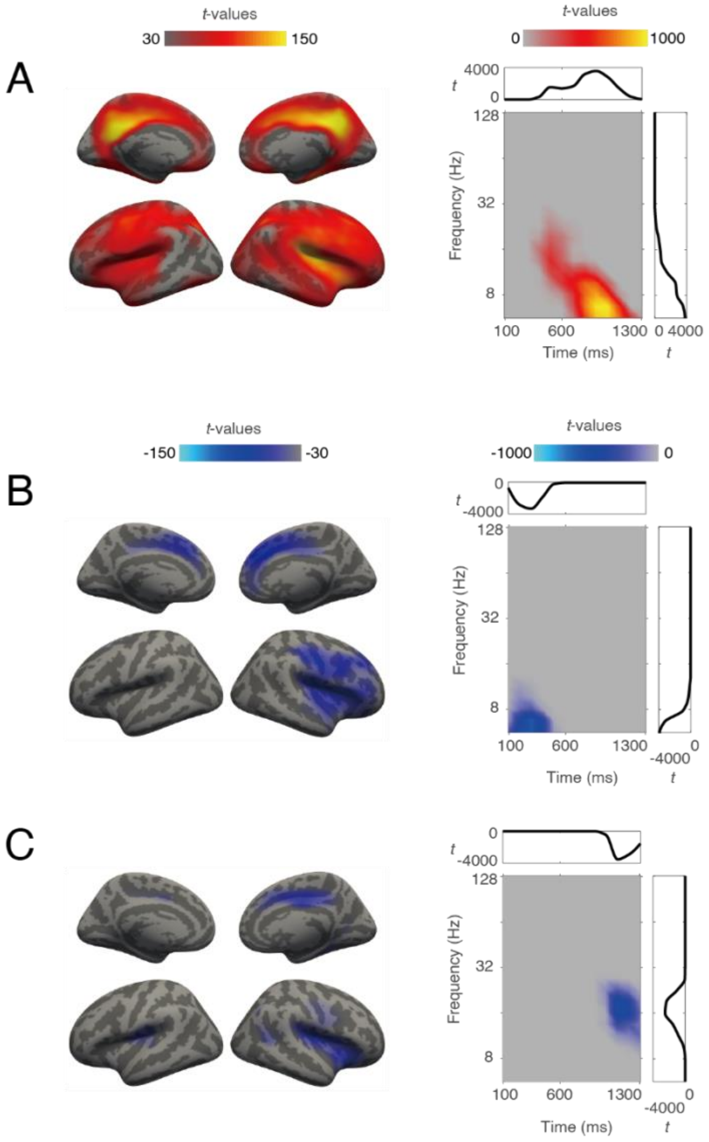

Fig. S3. Cluster analysis of power during response segments. (A - C). In each panel, both spatial (left) and time-frequency (right) distribution are illustrated. Color indicates accumulated *t* values. Side plots in the box are integrated *t* values against time (top, x-axis: time in ms, y-axis: *t* value) or frequency (right, x-axis: *t* value, y-axis: frequency in Hz).

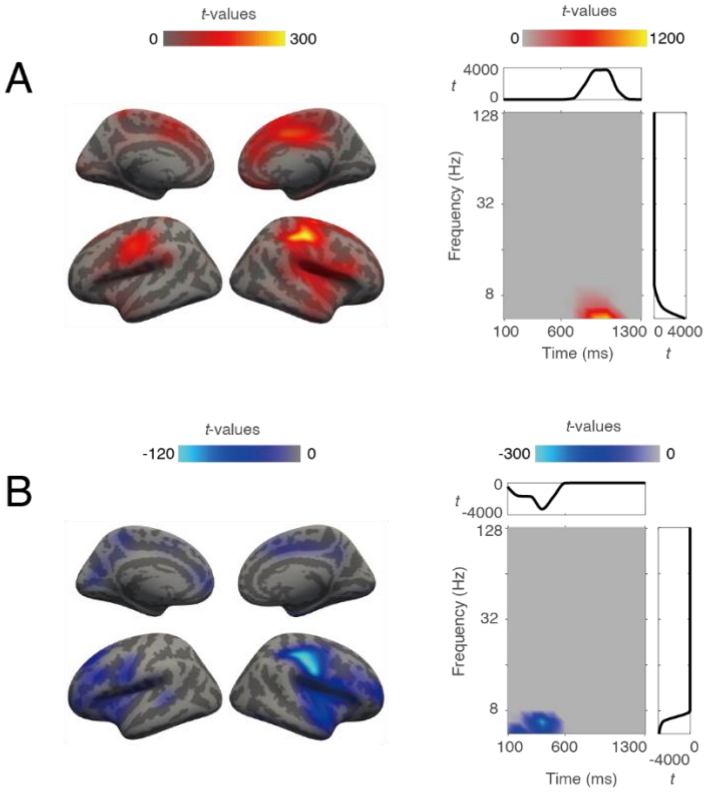

Fig. S4. Cluster analysis of coupling during response segments. In each panel, both spatial (left) and time-frequency (right) distribution are illustrated. Color indicates accumulated *t* values. Side plots in the box are integrated *t* values against time (top, x-axis: time in ms, y-axis: *t* value) or frequency (right, x-axis: *t* value, y-axis: frequency in Hz).
